# Environmental exposure pathways of microplastics and their toxic effects on ecosystems and the nervous system

**DOI:** 10.3389/ftox.2025.1649282

**Published:** 2025-09-19

**Authors:** Kuo Lu, Yixiang Que, Longfei Wang, Yanfan Wang, Jinyan Qiu, Yangyang Jia, Cong Ding, Dazhong Wang, Weyland Cheng, Yaodong Zhang

**Affiliations:** ^1^ Henan International Joint Laboratory of Children’s Infectious Diseases, Henan Children’s Hospital Zhengzhou Children’s Hospital, Children’s Hospital Affiliated to Zhengzhou University, Zhengzhou, China; ^2^ Children’s Hospital Affiliated to Zhengzhou University, Zhengzhou University, Zhengzhou, China; ^3^ Department of Pharmacy, Guangzhou Conghua District Hospital of Traditional Chinese Medicine, Guangzhou, Guangdong, China; ^4^ Institute of Children’s Health, Henan Academy of Innovations in Medical Science, Zhengzhou, China

**Keywords:** microplastic uptake, biobehavioral effects, neurodevelopment, ecosystem balance, nervous system mechanisms

## Abstract

Microplastics are a troubling consequence of modern civilization, permeating ecosystems worldwide and posing a risk to both the environment and human health. As studies have revealed their extensive distribution throughout bodies of water, soil, and the atmosphere, the ecological crisis and health issues linked to microplastics have become a significant concern within the global scientific community. These tiny particles can enter the human body through various routes, including ingestion, inhalation, and even skin contact, and they have been shown to cross critical barriers such as the placental and blood–brain barriers. Their accumulation in the food chain disrupts the delicate balance of ecosystems and may impair cognitive function and behavioral patterns in living organisms. Alarmingly, there is increasing evidence suggesting that microscopic particles may contribute to the increasing rates of neurodegenerative diseases. This paper reviews the pathways through which microplastics are ingested, their effects on biological functions, and the potential mechanisms that contribute to their neurotoxicity. We emphasize the urgent need for further research to elucidate the toxicological impacts of microplastics and devise effective strategies for mitigating their effects on both ecosystems and human health.

## 1 Introduction

Microplastics are plastic particles with a diameter of less than 5 mm that are widely found in the environment. With the increase in the use of plastics, the issue of microplastic pollution has garnered global concern. Microplastics disrupt the balance of ecosystems and pose risks to human health. Studies have shown that microplastics can be ingested by humans through a variety of pathways and may result in a range of health problems, including neurological damage (Le et al., 2024).

The distribution and concentration of microplastics vary significantly in different environmental settings. It has been found that microplastics are prevalent in a wide range of environments, including marine, freshwater, and soil, and the concentrations of microplastics are usually higher in urban and industrial areas than in remote areas (Wang B. et al., 2023). Microplastics in water not only threaten the survival of aquatic organisms but may also affect human health through bioaccumulation and biomagnification. Microplastics can also act as carriers for pollutants (including organic pollutants and heavy metals), increasing their bioavailability and amplifying their risks to ecosystems and human health (Sofield et al., 2024). There is growing evidence that microplastics have entered the human body. Microplastics have been found in alveolar lavage (Qiu et al., 2023) and sputum (Huang et al., 2022), suggesting that inhalation is one of the pathways by which microplastics enter the human body. Subsequently, microplastics have been detected in a variety of human tissues and body fluids, including lung tissue (Amato-Lourenco et al., 2021), liver (Horvatits et al., 2022), blood (S et al., 2024), pleural fluid (Guan et al., 2023), testes/seminal fluid (Zhao et al., 2023), and placenta/amniotic fluid (Halfar et al., 2023), suggesting that microplastics can traverse epithelial barriers, including the placental barrier. Recent studies have found microplastics in the human olfactory bulb, indicating a potential pathway for their translocation to the brain (Amato-Lourenco et al., 2024). These findings suggest an urgent need for further research into the health effects of microplastic exposure, particularly with regard to neurotoxicity and the potential for microplastics to breach the blood–brain barrier.

The health hazards associated with microplastics have attracted increasing scientific attention. When ingested, microplastics can disrupt the intestinal epithelial barrier, leading to gastrointestinal inflammation and gut microbiota dysbiosis. Once these particles cross the epithelial barrier, they can enter the bloodstream and accumulate in distant organs such as the liver and testes, potentially causing damage to these tissues. Inhaled microplastics can damage alveolar epithelial cells, trigger inflammation, and may contribute to respiratory conditions such as asthma (Lu et al., 2021) and pulmonary fibrosis (Song et al., 2024). Although most ingested microplastics are expelled through feces, the risks posed by residual particles that remain in the body should not be underestimated. Alarmingly, microplastics may penetrate the blood–brain barrier and the placental barrier, raising concerns about their effects on the central nervous system and the health of offspring. Studies indicate that ingesting microplastics could lead to neurotoxicity, impacting neuronal development, function, cognition, and behavior (Tian et al., 2024). Despite these findings, research on the neurotoxicity of microplastics, particularly regarding their effects on neurodevelopment and the nervous system, remains limited. The present paper aims to review the pathways through which microplastics are ingested, their effects on neurodevelopment and the nervous system, and the potential underlying mechanisms while underscoring the associated health risks and guiding future research efforts.

## 2 Intake pathways of microplastics and biological enrichment

### 2.1 Microplastics in the diet

Microplastics are prevalent in oceans, rivers, and soils, where they are ingested by a variety of organisms, leading to their accumulation in the food chain and posing significant health risks to humans (Tian et al., 2024). Fish and shellfish ingest microplastics, which can disrupt their biological functions and subsequently enter the human body through seafood consumption (Li et al., 2023). Furthermore, microplastics present in soil can be taken up by vegetables, resulting in human ingestion through our diets (Yang et al., 2024). Drinking water also serves as a major source of microplastics, primarily due to insufficient treatment processes that fail to remove these tiny particles (Sun and Wang, 2023). Additionally, plastic utensils can also release microplastics during use, contributing to dietary exposure. For example, plastic-containing tea bags have been found to release microplastics, raising concerns about systemic absorption and their impact on human health (Banaei et al., 2024; Sofield et al., 2024; Tilves et al., 2023).

### 2.2 Airborne microplastic inhalation

Airborne microplastics are a significant route of exposure, particularly in urban environments such as Beijing and London, where indoor concentrations are notably higher due to the degradation of various materials (Li et al., 2020; Liu et al., 2019; Wright et al., 2020). These microplastics can be inhaled, and studies have detected them in the nasal fluid of both indoor and outdoor workers, with indoor workers exhibiting a greater presence of these particles (Jiang et al., 2022). Further analysis of bronchoalveolar lavage fluid has identified 13 different types of microplastics in non-smokers, suggesting that these particles can penetrate deeply into the respiratory system (Qiu et al., 2023). Additionally, examinations of human lung tissue have revealed that microplastic concentrations are higher in the lower regions of the lung, which may increase the risk of respiratory damage and developing asthma (Jenner et al., 2022). Chronic exposure to these plastic particles, even at low concentrations, has been linked to airway inflammation and respiratory distress, highlighting the potential health risks associated with microplastic inhalation (Prata, 2018).

### 2.3 Personal care products and iatrogenic exposure

Microplastics present in personal care products are another prominent source of human exposure, with potential health risks including skin irritation and allergic reactions (Li et al., 2023). Experimental studies suggest that microplastics may promote the growth of skin cancer cells while impairing the function of normal skin cells (Wang Y. et al., 2023). Although limited studies have confirmed that microplastics can penetrate the skin barrier, there is a pressing need for further research to understand their systemic toxicity (Liu and You, 2023). Moreover, medical interventions present another critical route of exposure as microplastics have been detected in infusion containers and shown to enter systemic circulation through intravenous injections, raising serious health concerns (Zhu et al., 2023). There is an urgent need for monitoring and risk management in the medical field, particularly because microplastics can cross the placental barrier, potentially impacting fetal brain tissue and leading to neurodevelopmental issues (Li et al., 2023; Bongaerts et al., 2022).

**FIGURE 1:**
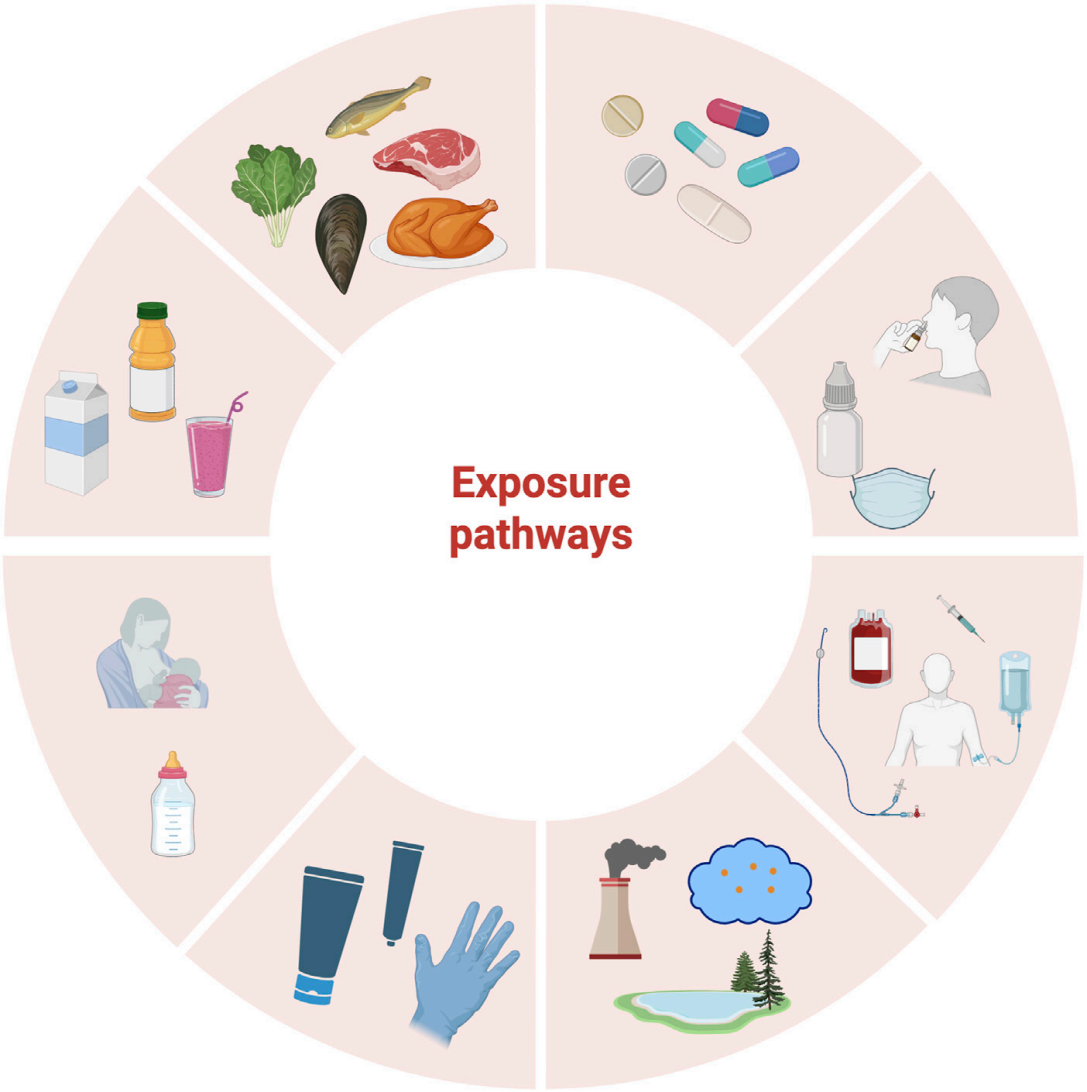
Possible pathways of human exposure to microplastics: through foods, such as fish, meat, poultry, vegetables, and shellfish, and through beverages, such as boxed milk, bottled beverages, and cupped beverages. Exposure through maternal and infant routes, such as breastfeeding or bottle-feeding; exposure through personal care products such as hand cream, cosmetics, and gloves; exposure through environmental media such as air (e.g., clouds) and aquatic systems (e.g., lakes); exposure through medical devices (such as syringes, infusion bags, and blood bags), eye and nasal medications (such as eye drops, nasal drops, and inhalers), and oral medications (such as tablets and capsules).

As illustrated in Figure 1, humans can be simultaneously exposed to microplastics through various pathways, complicating the task of identifying the exact sources of microplastics that accumulate within the body. Current technological limitations, particularly in detecting microplastics in living organisms, hinder our ability to fully evaluate exposure profiles. Additionally, there is a considerable gap in research regarding how factors such as particle size, shape, and chemical properties of microplastics influence their bioavailability, deposition efficiency, and ability to traverse physiological barriers, including the epithelial barrier, blood–brain barrier, and placental barrier. Furthermore, there is currently insufficient epidemiological evidence linking microplastic exposure to specific diseases.

## 3 Impacts of microplastics on ecosystems, behavior and cognitive functions

### 3.1 Behavioral toxicity of microplastics to different organisms

Microplastic exposure in various biological models leads to notable behavioral changes, such as decreased locomotion in *Caenorhabditis elegans* (Chen et al., 2024), reduced burrowing and feeding in bivalves (Zhang et al., 2025), and inhibited swimming in carps, indicating potential interference with nervous and metabolic functions (Chen et al., 2022). Additionally, birds exposed to microplastics exhibit reduced social interactions and altered foraging behaviors, which can significantly affect their social dynamics (Takai et al., 2022; Tamura et al., 2024). In mammals, microplastics disrupt social structures, leading to behavioral changes that reduce social tendencies and increase anxiety, thereby weakening the cohesion of social groups (Trivedi et al., 2025; Wang L. et al., 2024). Furthermore, microplastics influence food choices and foraging behaviors, which can alter roles within groups and ultimately affect survival, reproduction, and ecological adaptability (Pollack et al., 2025). The impact of microplastic exposure on behavior varies with the age of the organisms. For example, larval zebrafish experience a decrease in swimming ability, while adult zebrafish display hyperactivity (Qiang and Cheng, 2019; Chen et al., 2020). Older marine species, such as Alaskan pollock, tend to ingest more microplastics, correlating with their age and suggesting cumulative effects on their growth and reproductive capabilities (Ding et al., 2023). In yellow mealworms, those that are 3 months old show the greatest ability to degrade and tolerate microplastics, while both younger and older mealworms encounter higher rates of mortality and developmental challenges (Zhong et al., 2023). This highlights the critical role of age in determining toxic responses to microplastic exposure.

However, laboratory research often involves high-dose artificial microplastic interventions, which can lead to results that may not accurately reflect real-world conditions, as the concentration of microplastics found in natural environments is typically much lower than that used in laboratory settings. Additionally, there is a notable gap in research concerning more advanced organisms, such as primates, which limits our understanding of how microplastics may affect ecological behavioral cognition. Therefore, further exploration and investigation by researchers are essential to uncover the true impacts of microplastics on these complex organisms and their behaviors in the wild.

### 3.2 Impact of microplastics on cognitive functions

Exposure to microplastics has been linked to diminished learning and memory capabilities, particularly during crucial stages of neurodevelopment, with potential long-term effects on cognitive function (Oger et al., 2024). This environmental pollution not only endangers ecosystems but also poses immense risks to human health and the development of children, highlighting the urgent need for action. The neurotoxic effects of microplastics have been closely connected to the onset of various neurodegenerative diseases. Studies indicate that microplastics may accelerate the progression of conditions such as Alzheimer’s and Parkinson’s diseases by instigating neuroinflammation and oxidative stress (Gou et al., 2024). For instance, the ingestion of microplastics may lead to neuronal dysfunction and cell death, which are hallmark characteristics of neurodegenerative disorders (Sun et al., 2024). Moreover, microplastics may also affect the gut–brain axis by altering the gut microbiota composition, potentially influencing the development of neurodegenerative diseases (Tong et al., 2022). These findings underscore the serious threat posed by microplastics as environmental pollutants to human health, particularly concerning neurological wellbeing, and suggest a pressing need for enhanced research and monitoring of microplastics and their health implications.

### 3.3 Ecosystem-level impacts of microplastics

Microplastics not only impact individual organisms but also disrupt the balance of entire ecosystems by altering interactions among species. For instance, research on Daphnia magna has shown that exposure to microplastics can weaken its defenses against predators, which include changes in morphology, reproduction, and behavior (Wang Z. et al., 2023). This reduction in defensive capabilities may make Daphnia magna more vulnerable to predation. Furthermore, smaller microplastics have a more pronounced effect on these behavioral defenses than larger microplastics, highlighting the critical role of particle size in the ecological consequences of microplastics. Additionally, chemical signals from fish significantly influence the effects of microplastics on the behavior of Daphnia magna. Although microplastics alone do not significantly alter the vertical distribution of Daphnia magna, their daily average depth of migration becomes notably shallower when fish chemical signals are present (Wang Z. et al., 2023). This indicates that microplastics may disrupt biotic interactions and behavioral strategies by adsorbing or interfering with these chemical signals.

## 4 Influence of microplastic properties on neurotoxicity

### 4.1 Concentration-dependent toxic effects of microplastics

The concentration range of microplastics in the environment plays a critical role in determining the exposure levels and health impacts on various organisms. High concentrations of microplastics, particularly polystyrene, induces acute toxic effects, as evidenced by behavioral changes in zebrafish, which include reduced swimming patterns and activity levels, alongside neurotoxic signs such as alterations in neurotransmitter levels and cellular damage (Vercauteren et al., 2024; Xie et al., 2024). Conversely, the chronic toxic effects associated with low concentrations of microplastics warrant careful consideration, especially regarding long-term exposure. Research indicates that zebrafish exposed to low concentrations of microplastics experience downregulation of immune-related genes and disruptions in their activity rhythms, even in the absence of immediate physiological damage (Limonta et al., 2019). Over time, these low concentrations may impair cognitive functions by negatively affecting the nervous system, leading to potential deficits in memory and learning abilities after prolonged exposure (Revel et al., 2020). Furthermore, sensitivity to microplastic concentrations varies significantly among different species, influenced by their physiological traits, ecological roles, and behavioral patterns. For example, aquatic invertebrates such as water fleas and mollusks demonstrate considerable physiological stress at lower microplastic concentrations, while fish typically exhibit toxic responses only at higher levels (Liang et al., 2024; Sun et al., 2021). This variability underscores the importance of ecological adaptability and physiological structure in determining species sensitivity to microplastics. However, establishing concentration thresholds for microplastics presents numerous challenges in experimental design and methodology, primarily due to the diverse nature of microplastics and the intricate ways they interact with biological systems (Bai et al., 2021; Balzani et al., 2022).

### 4.2 Microplastic size and shape are key factors in cellular uptake, distribution and clearance

The size of microplastics plays a crucial role in their absorption, distribution, and clearance within organisms. Smaller microplastics can easily enter cells and interact with cellular structures, triggering toxic responses such as oxidative stress and inflammation. These reactions may have adverse effects on the cognitive functions of organisms. For example, studies on Pacific oyster (Haliotis dactyla) larvae have shown that the effects of high-density polyethylene microplastics on their development and swimming behavior are influenced by particle size (Bringer et al., 2020). Smaller microplastics tend to disrupt organismal behavior more significantly, possibly because they are more easily ingested and can accumulate within the body, thereby exerting more direct effects on physiological functions. The size and nature of particles influence their uptake efficiency via endocytosis or phagocytosis mechanisms. Particles larger than 0.5 μm are typically taken up via phagocytosis, while those smaller than this size are more likely to be absorbed by cells via endocytosis (Hallett, 2020). The size of microplastics also affects their ability to cross the blood–brain barrier. Studies have shown that smaller microplastics, particularly those at the nanoscale, are more likely to penetrate this barrier. When microplastics accumulate in brain tissue, they can cause neuroinflammation and cellular damage, potentially leading to cognitive dysfunction (Barrick et al., 2024). For example, a study involving mice showed that long-term exposure to polystyrene microplastics led to inflammatory responses and cognitive decline, highlighting the close link between microplastic size and their neurotoxic effects (Jin et al., 2022). Particles of different shapes exhibit notable differences in cellular uptake efficiency. Generally, spherical particles are more easily taken up by cells due to their symmetry and uniformity. Rod-shaped particles, however, may exhibit higher uptake rates under certain conditions (Ogino et al., 2021), particularly when actively phagocytosed by cells (Cao et al., 2022), possibly due to changes in cell membrane structure and activation of intracellular signaling pathways (Roy et al., 2024).

The systemic distribution of particulate matter and the transport of immune cells are closely related to interactions with serum proteins. Macrophages and lymphocytes play important roles in the systemic distribution of particulate matter. Macrophages effectively phagocytose particulate matter and interact with the lymphatic system to transport the phagocytosed particulate matter to lymph nodes, thereby clearing particulate matter from the body (Jinarat et al., 2025). The uptake of particulate matter can activate macrophages, leading to the secretion of pro-inflammatory cytokines and triggering an inflammatory response (Hallett, 2020). Additionally, particulate matter can promote T-cell activation and proliferation, enhancing the body’s immune response to particulate matter (Jinarat et al., 2025). Furthermore, lymphocytes regulate macrophage function by releasing cytokines, forming a complex immune network that is crucial for clearing particulate matter from the body and maintaining immune homeostasis (Li et al., 2022). Serum proteins (such as albumin, globulin, and fibrinogen) play a key role in the systemic distribution of particulate matter. Serum proteins can bind to particulate matter to form a protein corona, a process that influences the biocompatibility and toxicity of particulate matter (Ju et al., 2022). Fibrinogen in serum can promote phagocytosis by macrophages by binding to particulate matter, thereby enhancing the immune response (Mamidala et al., 2022). Additionally, the surface properties of particles (such as charge and hydrophilicity) influence macrophage endocytosis and phagocytosis efficiency, along with their binding capacity with serum proteins, thereby affecting their distribution and clearance rates within the body (Yang et al., 2023).

### 4.3 Synergistic/antagonistic effects of compound pollution on behavior and cognition

The effects of compound pollution, particularly the synergistic and antagonistic interactions among various pollutants, have garnered significant attention in the field of ecotoxicology. When microplastics are present alongside other pollutants such as heavy metals and organic substances, their influence on biological behavior and cognitive functions can differ markedly compared to individual pollutants. For example, studies have indicated that the combination of microplastics and antibiotics can lead to complex synergistic effects, which may alter the behavioral patterns and neurotransmitter levels in aquatic organisms (Wang Y. et al., 2024). Furthermore, the presence of microplastics can exacerbate the toxicity of other pollutants. Research shows that heavy metals become significantly more toxic when adsorbed onto microplastics, leading to heightened oxidative stress within affected organisms (Gao et al., 2025). This combined pollution can disrupt neurotransmitter balance, ultimately impacting cognitive functions and behavioral performance. Experimental evidence suggests that the interaction between microplastics and heavy metals can further compromise the survival and functionality of nerve cells by intensifying oxidative stress responses (Liu F. et al., 2024). Therefore, investigating how microplastics interact with other pollutants and their subsequent effects on biological behavior and cognitive functions is crucial for understanding the complexities of pollution in ecosystems. This knowledge also lays the groundwork for developing effective environmental protection measures and public health strategies in the future.

### 4.4 Polymer types, additives and plasticizers

Common additives in microplastics, including phthalates and bisphenol A, can leach into the environment and cause toxic effects in various organisms. These additives are particularly concerning due to their interference with the neuroendocrine system, which plays a crucial role in regulating physiological functions. Research has shown that phthalates can disrupt endocrine functions, resulting in altered physiological behaviors in affected organisms (Meera et al., 2023). Furthermore, studies indicate that when microplastics are present alongside these harmful additives, they can exhibit synergistic toxicity, which intensifies the detrimental effects on aquatic life (Kasmuri et al., 2022).

## 5 Neurodevelopmental effects of microplastics

**FIGURE 2:**
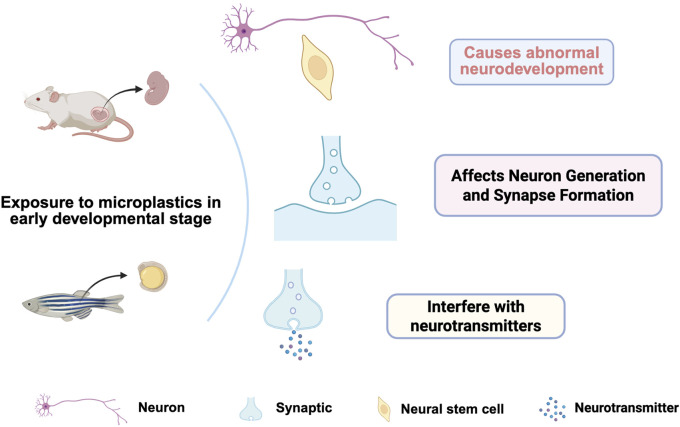
Influence of microplastics on early neural development. In the early stages of development, exposure to microplastics may cause abnormal neurodevelopment, alter the formation of neurons and synapses, and interfere with neurotransmitters.

As emerging environmental pollutants, microplastics have gained increased attention for their undesirable effects on living organisms. Studies have shown that organisms at early developmental stages exhibit high sensitivity to microplastic exposure. During pregnancy and lactation, maternal exposure to microplastics may profoundly affect the neurodevelopment of the offspring (Figure 2). For instance, one study found that the offspring of female rats exposed to polystyrene nanoplastics during gestation and lactation showed abnormalities in cortical thickness and neuronal migration, characterized by increased proliferation of cortical cells and decreased numbers of deep neurons (Tian et al., 2024). In addition, the presence of microplastics may also lead to disturbances in neuron generation and synapse formation, thereby affecting behavioral and cognitive functions (Gupta et al., 2024). Thus, organisms in early developmental stages are profoundly vulnerable to microplastic exposure, emphasizing the importance of incorporating this risk into environmental protection strategies and public health policy development.

### 5.1 Neurodevelopmental abnormalities

Exposure to microplastics is strongly associated with neurodevelopmental abnormalities (Figure 2). Maternal exposure to microplastics during pregnancy has been found to result in neurodevelopmental disorders in offspring, as evidenced by morphological changes and functional defects in neurons (Santoro et al., 2024). For example, maternal exposure to polystyrene nanoplastics (PSNPs) causes developmental abnormalities in the embryonic brain, affecting neural stem cell function and neuron production (Jeong et al., 2022). The mechanism of this developmental abnormality may be related to the interference of microplastics with neurotransmitters and the effects on the expression of neurodevelopment-related genes, such as the inhibition of the brain-derived neurotrophic factor (BDNF) signaling pathway (Hong et al., 2021). Exposure to microplastics inhibits neuronal development, affecting their ability to proliferate and migrate. Studies have shown that neurons exposed to microplastics exhibit significant inhibition of growth and differentiation, resulting in a reduced number of neurons and abnormal morphology (Shi et al., 2023). Prenatal exposure to PS-NPs caused a reduction in cortical thickness and an increase in cortical cell proliferation in fetal rats. In addition, the offspring showed disturbances in neocortical migration, evidenced by an increase in the proliferation of superficial neurons and a decrease in the number of deep neurons (Tian et al., 2024). Moreover, the effects of microplastics are also observed in the interference with key signaling pathways that are essential for normal neuronal development (Wang J. et al., 2023). Thus, environmental contamination by microplastics poses a threat not only to ecosystems but also potentially exerts far-reaching effects on the development and function of the nervous system.

### 5.2 Effects on neuron generation and synapse formation

The effects of microplastics on neurogenesis and synapse formation have been confirmed by several studies. Experimental results show that neural stem cells exposed to microplastics develop marked defects in proliferation and differentiation. Exposure to polystyrene microplastics caused significantly wider synaptic gaps and reduced postsynaptic density in mouse hippocampal synapses, which may be related to microplastic-induced oxidative stress and apoptosis (Tian et al., 2024). Additionally, the presence of microplastics may further affect synapse formation and function by altering neurotransmitter levels and the efficiency of nerve conduction (Im et al., 2022). These findings suggest that microplastics not only have a direct effect on neuron production but may also contribute to long-term cognitive and behavioral problems by affecting synapse formation and function.

### 5.3 Neurotransmitter interference

The presence of microplastics has a substantial impact on the normal function of neurotransmitters. Studies have shown that exposure to microplastics leads to disruption of neurotransmitter release and metabolic processes. For example, motor neurodevelopment in zebrafish was considerably affected after exposure to microplastics, as evidenced by altered neurotransmitter levels (such as acetylcholine, norepinephrine, and dopamine) and enzyme activity (AChE, ChAT, and ChE) (Li X. et al., 2024). These changes affect neuronal signaling and may also result in behavioral abnormalities, such as hyperactive swimming behavior, suggesting that microplastics disrupt normal nervous system function by altering the balance of neurotransmitters (Jeong et al., 2022). In addition, photoaging of microplastics has been found to exacerbate this neurotransmitter interference, further affecting neurodevelopment and behavioral performance (Li T. et al., 2024).

## 6 Possible mechanisms by which microplastics affect the nervous system

### 6.1 Inhibition of AChE activity

**FIGURE 3:**
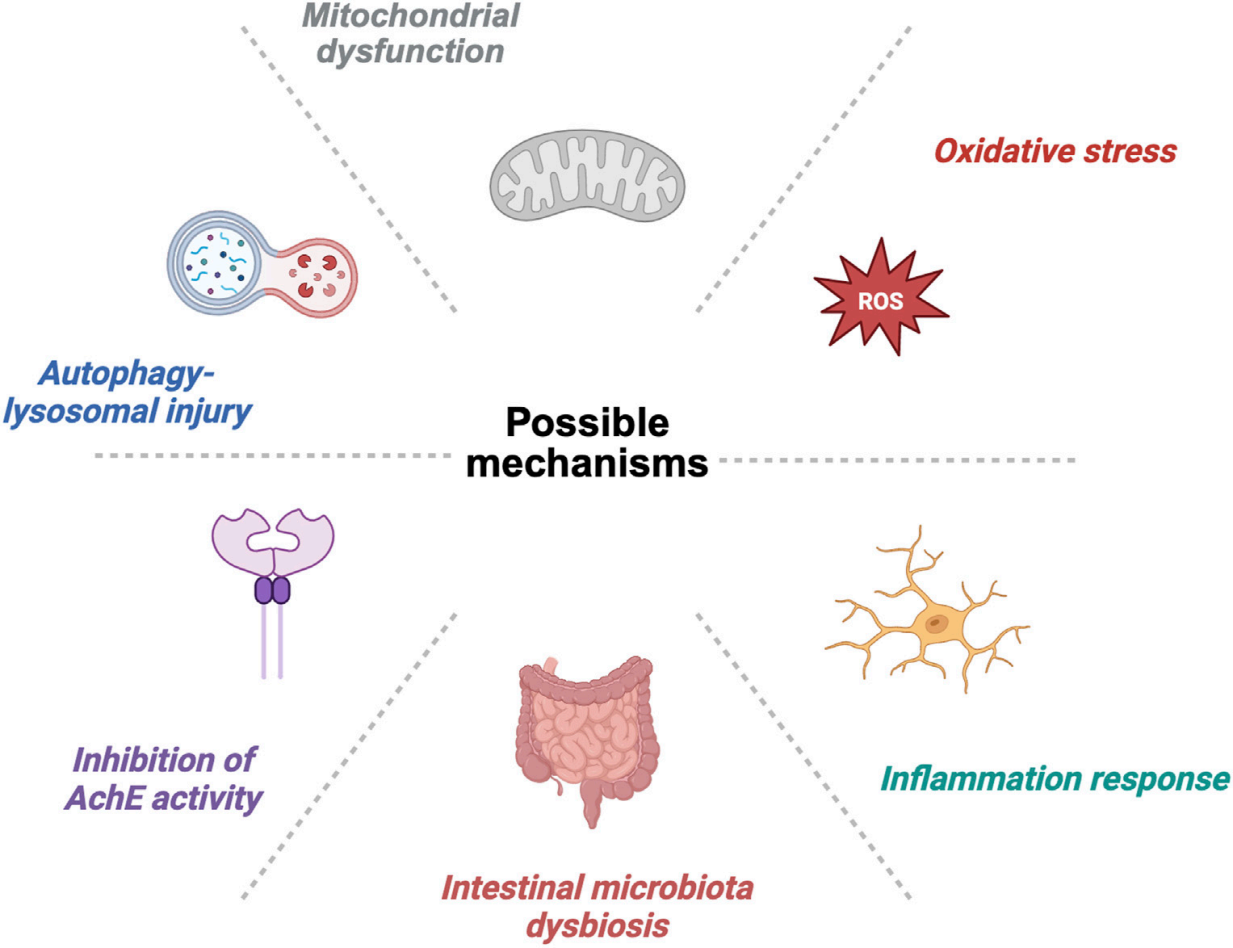
Possible mechanisms by which microplastics affect neural development and the nervous system.

One mechanism by which microplastics affect the nervous system is the inhibition of acetylcholinesterase (AChE) activity, an enzyme that plays an important role in neurotransmission by breaking down the neurotransmitter acetylcholine, which regulates nerve signaling (Figure 3). Studies have shown that exposure to microplastics may lead to a decrease in AChE activity, resulting in the accumulation of acetylcholine and potentially triggering a variety of pathological changes in the nervous system, such as cognitive impairment and behavioral abnormalities (Sun et al., 2024). In addition, the inhibitory effect of microplastics on AChE may be influenced by their chemical composition and physical properties, which can affect their cellular uptake and metabolism in organisms, thereby further exacerbating neurotoxicity (Santoro et al., 2024). Therefore, it is essential to understand the mechanisms by which microplastics affect AChE activity in order to assess their potential harm and associated risks to the nervous system.

### 6.2 Inflammatory response

Exposure to microplastics may also trigger an inflammatory response in the nervous system. It has been found that microplastics can lead to neuroinflammation by activating immune cells and releasing inflammatory factors (Sofield et al., 2024). Microplastic exposure caused a marked upregulation of pro-inflammatory cytokines IL-6 and IL-1β in the zebrafish brain, and *in vitro* experiments with HMC3 microglia further supported this finding (Yang et al., 2025). Studies in human and animal models have demonstrated that neuroinflammation is closely related to mood disorders (Guo et al., 2023; Miller and Raison, 2016). This inflammatory response not only damages nerve cells but may also lead to neurological dysfunction, which, in turn, affects cognitive ability and behavioral performance. The presence of microplastics may further exacerbate the inflammatory response in the nervous system by affecting gut microbiota composition and gut–brain axis function (Tong et al., 2022). Therefore, exploring the mechanisms of microplastic-induced inflammation could help understand its long-term effects on the nervous system.

### 6.3 Oxidative stress

Oxidative stress is another important mechanism by which microplastics affect the nervous system (Figure 3). The intake and accumulation of microplastics may lead to an increase in the production of reactive oxygen species (ROS) in the body, which triggers oxidative stress (Lin et al., 2025). Oxidative stress not only damages cell membranes and DNA but may also lead to apoptosis and neuronal death, which is particularly evident in neurodegenerative diseases (Santoro et al., 2024). Studies have shown that microplastics can elevate oxidative stress levels, which may be closely associated with the development of a variety of neurological diseases, particularly in the elderly population (Savuca et al., 2024). Therefore, an in-depth study into the mechanisms by which microplastics induce oxidative stress is essential for the development of interventions targeting neuroprotection.

### 6.4 Mitochondrial dysfunction

Exposure to microplastics may lead to mitochondrial dysfunction, which is particularly important in nerve cells. Mitochondria are the center of cellular energy metabolism, and their dysfunction can directly affect the survival and function of nerve cells. *In vitro* experiments on SH-SY5Y cells demonstrated that PS-NPs induced changes in mitochondrial structure, decreased mitochondrial membrane potential, and reduced ATP production (Huang et al., 2023). Further studies have found that high concentrations (100 mg/L) of PS-NPs caused mitochondrial dysfunction, increased intracellular Ca^2+^ levels, decreased mitochondrial membrane potential and ATP levels, and produced neurotoxic effects. Another *in vivo* study on zebrafish also found mitochondrial damage in the brain exposed to microplastics, manifested as decreased mitochondrial membrane potential, reduced mtDNA copy number, and altered mRNA expression levels of genes related to mitochondrial biosynthesis, fusion, and division in the brain (Zhang et al., 2023). Furthermore, mitochondrial dysfunction may also be associated with neuroinflammation and neurodegeneration, which further exacerbates the toxic effects of microplastics on the nervous system (Sun et al., 2024). Therefore, exploring the mechanisms by which microplastics affect mitochondrial function is critical to understanding their role in neurotoxicity.

### 6.5 Autophagy–lysosomal injury

Microplastics may also affect nervous system health by impairing the autophagy–lysosomal pathway. Autophagy is an important cellular mechanism for removing damaged and redundant organelles, with lysosomes playing a key role in this process. Dysfunction of the autophagy–lysosomal system may also lead to the accumulation of harmful substances in neuronal cells, potentially contributing to the onset and progression of neurodegenerative diseases (Chularojanamontri et al., 2024). The recovery of autophagy–lysosomal system function plays an important role in the improvement of traumatic brain injury and ischemia–reperfusion injury-induced brain injury (Liu H. et al., 2024; Woo et al., 2025). Studies have shown that exposure to microplastics may lead to autophagy dysfunction, which, in turn, affects intracellular metabolic processes and clearance pathways (Fang et al., 2025). However, whether microplastic exposure affects behavior and neurological function through the autophagy–lysosomal pathway still requires further research for verification. Therefore, investigating the effects of microplastics on the autophagy–lysosome pathway can help reveal potential neurotoxic effects.

### 6.6 Dysbiosis of the intestinal microbiota

In recent years, studies have shown that microplastics can adversely affect the nervous system by disrupting the intestinal microbial community (Fulling et al., 2019). Upon entering the gut, microplastics can alter the composition and function of gut microbes. They can adhere to the surface of microorganisms and interfere with their metabolic activities, leading to imbalances in the microbial community. This imbalance disrupts gut homeostasis, triggers an inflammatory response, increases intestinal permeability, and can facilitate the entry of harmful substances into circulation (Pacher-Deutsch et al., 2025). There is a strong connection between gut microbes and the nervous system, known as the gut–brain axis. Disruptions in the gut microbiota can influence the nervous system through multiple mechanisms. First, disturbed gut microbes release abnormal metabolites that can reach the brain through the bloodstream and interfere with the synthesis and release of neurotransmitters. For example, certain gut microbes are capable of synthesizing neurotransmitters or their precursors, and when the microbial community is imbalanced, the levels of these neurotransmitters may be altered, thereby affecting the normal function of the nervous system. Second, gut inflammation activates the immune system to release inflammatory cytokines. These inflammatory factors can cross the blood–brain barrier, inducing neuroinflammation and causing neuronal damage. Additionally, gut microbial disorders can impair the gut barrier, allowing harmful substances to enter the bloodstream and further damage the nervous system.

## 7 Future research directions and public health recommendations

As microplastic contamination continues to increase, it is essential to use advanced research methods, such as high-throughput sequencing and nanotechnology, for effective detection and analysis. These innovative techniques greatly increase sensitivity in detecting microplastics and enhance understanding of their distribution and toxicity, shedding light on their impact on both health and ecosystems (Zhang et al., 2024; Gupta et al., 2024). In response to this growing concern, policymakers should strengthen regulations on plastic use, promote the adoption of sustainable materials, and raise public awareness to encourage behavioral changes that support effective policy development (Wang M. et al., 2024). Strategies aimed at preventing microplastic exposure should prioritize source control by minimizing plastic consumption and encouraging the use of biodegradable alternatives. Additionally, individuals can reduce exposure through increased awareness and proactive measures, such as choosing plastic-free packaging. To ensure timely health interventions, it is also crucial to establish monitoring systems that can accurately assess microplastic levels in water and food.

In conclusion, microplastics are widely found in the environment and have the potential to interfere with the development of the nervous system, primarily through mechanisms such as oxidative stress and inflammation. However, the research findings on their impact are inconsistent, and most studies focus on experimental animal models such as mice and zebrafish, indicating that further research is needed. Future studies should aim to standardize methodologies to enable comparison of results across different research efforts. Epidemiological investigations into microplastic exposure should also be expanded to systematically assess potential association with human health, along with the dose-response relationship. In addition, governments must implement regulations that limit emissions of microplastics, promote the use of biodegradable materials, and enhance public awareness regarding the issue. Furthermore, international collaboration is essential for gaining a comprehensive understanding of the hazards posed by microplastics and developing effective protective measures, with the overarching goal of safeguarding public health and protecting the environment.
